# Prognostic Role of Systemic Inflammatory Markers in Patients Undergoing Surgical Resection for Oral Squamous Cell Carcinoma

**DOI:** 10.3390/biomedicines10061268

**Published:** 2022-05-29

**Authors:** Uiju Cho, Yeoun-Eun Sung, Min-Sik Kim, Youn-Soo Lee

**Affiliations:** 1 Department of Hospital Pathology, St. Vincent’s Hospital, College of Medicine, The Catholic University of Korea, Seoul 06591, Korea; hailtoya@catholic.ac.kr; 2 Department of Hospital Pathology, Seoul St. Mary’s Hospital, College of Medicine, The Catholic University of Korea, Seoul 06591, Korea; yesung@catholic.ac.kr; 3 Department of Otorhinolaryngology, Seoul St. Mary’s Hospital, College of Medicine, The Catholic University of Korea, Seoul 06591, Korea; entkms@catholic.ac.kr

**Keywords:** oral cancer, inflammation, prognosis, surgical resection, overall survival

## Abstract

Background: A high platelet–lymphocyte ratio (PLR) is a marker of systemic inflammation and, together with the neutrophil–lymphocyte ratio (NLR), is associated with poor outcomes in several cancers. We investigated the prognostic value of PLR and other systemic inflammatory markers, such as NLR, systemic immune-inflammation index (SII), and systemic inflammation response index (SIRI), in oral squamous cell carcinoma (OSCC) patients undergoing surgical resection. Methods: We derived PLR, NLR, SII, and SIRI from a retrospective chart review of 269 consecutive OSCC patients. The complete blood count examined in the immediate preoperative period was used to compute PLR, NLR, SII, and SIRI. We analyzed the relationship between these systemic inflammatory markers and the clinicopathologic characteristics, disease-specific survival (DSS), and progression-free survival (PFS) of patients. Results: In the univariate analysis, high PLR and SII were significantly associated with worse DSS and PFS (all *p* < 0.05). In the multivariate analysis, PLR (HR 2.36, 95% CI 1.28–4.36 for DSS; HR 1.80, 95% CI 1.06–3.06 for PFS) was an independent predictor of survival outcomes. When PLR was analyzed as a continuous variable, the relationship between the outcome and preoperative PLR was not monotonically linear. In the subgroup analysis, PLR was more strongly associated with DSS and PFS in patients who were male, had stage III/IV OSCC, or had lymph node metastasis. Conclusion: Our data suggest that in OSCC patients, the pretreatment PLR is an independent predictor of DSS and PFS. The PLR is a readily available biomarker that will improve prognostication and risk stratification in OSCC.

## 1. Introduction

Oral squamous cell carcinoma (OSCC) is a carcinoma with squamous differentiation arising from the mucosal epithelium of the oral cavity and mobile tongue. The global incidence of oral cancer, the majority being squamous cell carcinoma, is approximately 3.5 million new cases per year, and it causes 1.7 million deaths per year. Oral cancer accounts for 2.0% of all cancers [1]. In Korea, oral cancer is the second most common cancer among head and neck cancers, and the incidence of oral cancer has been slightly increasing in recent decades [2,3]. The incidence rate is especially rising more steeply in the third- or fourth-decade age groups [4]. Smoking, drinking alcohol, lifestyle changes, the popularization of early diagnosis, and genetic factors could be the causes of such an increase [4,5]. Oral cancer is more common among men than women, and most common in the fifth and sixth decades [6]. The survival rate of oral cancer is approximately 50% [7]. Advancements in traditional treatment modalities, i.e., surgery, chemotherapy, and radiotherapy, have not been able to noticeably increase the survival rate, yet the side effects of these treatments are significant.

Currently, the prediction of tumor progression or recurrence depends largely on classic histologic parameters, such as tumor size, depth of invasion, pattern of invasion, and nodal status [6]. Many novel biomarkers have been investigated to achieve better risk stratification for adjuvant treatment modalities or more aggressive treatment in patients with distant metastasis [8]. PD-L1 is a recently discovered prognostic biomarker and immune checkpoint inhibitor; therefore, anti-PD-L1 therapy would be a promising treatment for OSCC [9,10]. However, none of the novel biomarkers have been recommended as prognosticators valid for clinical use to date.

The cell-mediated inflammatory response has been shown to play a critical role in cancer development and growth. For example, tumor-associated neutrophils are considered potent stimulators of angiogenesis, and their protumoral cytokines promote tumor growth [11,12]. Additionally, extensive disruption of hematopoiesis occurs as cancer progresses [11,13]. Changes in the systemic inflammatory response to tumor cells, especially white blood cells and platelets, have drawn attention as valuable prognostic biomarkers [11]. The systemic inflammatory markers, i.e., neutrophil–lymphocyte ratio (NLR) and platelet–lymphocyte ratio (PLR), have been used as prognostic biomarkers in various types of cancers [14,15,16,17,18]. These serum inflammatory markers are easily available because they can be retrieved from routine blood tests [19].

Until now, the NLR, which uses differential white cell counts, has been the most extensively investigated marker in operable and inoperable cancers [19,20]. Elevated PLR and NLR were associated with poor survival outcomes in previous studies [21,22]. Furthermore, investigators explored the combination of the scores with acute-phase protein-based scores (Glasgow Prognostic Score) [23] or developed novel inflammatory markers to provide additional prognostic value in different cancers [24]. Among such novel markers, the most recently developed are the systemic immune-inflammation index (SII) [24,25] and systemic inflammation response index (SIRI) [26,27]. They are derived from three types of inflammatory cells (lymphocytes, neutrophils, and platelets or monocytes), and were shown to be independent predictors of overall survival in patients with lung [24,27], breast [26], esophageal [25], and urologic cancers [28]. High preoperative SII and SIRI were also shown to be independent prognostic factors in patients with OSCC, but the data are still very limited [29,30,31,32].

In OSCC, data to support the clinical value of different systemic inflammatory markers are still accumulating. However, the results are controversial and need further research [8,33,34]. Furthermore, the data to evaluate the clinical value of systemic inflammatory markers are still insufficient, since studies of inflammatory markers have focused on NLR, and no study has simultaneously compared the prognostic values of NLR, PLR, and the new emerging markers, SII and SIRI, in OSCC.

In this study, we aimed to validate the prognostic value of a panel of systemic inflammatory markers, NLR, PLR, SII and SIRI. In addition, we evaluated the relationships of clinicopathologic parameters and systemic inflammatory markers with survival in OSCC patients.

## 2. Materials and Methods

### 2.1. Study Population

We retrospectively identified and enrolled 269 patients with oral cavity and mobile tongue squamous cell carcinoma who had undergone surgical resection at Seoul St. Mary’s Hospital between January 2003 and December 2019. We excluded patients with other malignancies, with autoimmune diseases, who had received neoadjuvant therapy, or who had insufficient preoperative blood tests carried out to calculate systemic inflammation markers for the study.

This study was conducted in accordance with the amended Declaration of Helsinki. The study was approved by our Institutional Review Board (Seoul St. Mary’s Hospital, IRB No. 86651124), and the requirement for informed consent was waived by the Institutional Review Board.

### 2.2. Data Collection

The following data were collected from the patients’ medical records: date of the primary cancer diagnosis, age at diagnosis, anatomical sublocation, tumor size, tumor differentiation, depth of invasion, lymphatic invasion, vascular invasion, perineural invasion, presence of lymph node metastasis, distant metastases, and adjuvant therapy. M stage was defined as M0 unless distant metastasis was specified in the medical records. The slides were reviewed by an expert pathologist (S.Y.E.) and restaged according to the American Joint Committee on Cancer (AJCC) staging manual, 8th edition [35]. The differential white blood cell (WBC) count that was measured within one month before the surgery as part of the routine preoperative workup was collected from the medical report. Systemic inflammatory markers were defined as follows: NLR (absolute neutrophil count/absolute lymphocyte count), PLR (absolute platelet count/absolute lymphocyte count), SII (platelet count × neutrophil count/lymphocyte count), and SIRI (neutrophil count × monocyte count/lymphocyte count).

The cutoff values for platelet count, NLR, PLR, SII, and SIRI were determined from receiver operating characteristic (ROC) curves for overall survival considering both sensitivity and specificity. The cutoff values and the area under the curve (AUC) values are shown in Table 1. The patients were divided into two groups (the low group and high group) based on NLR, PLR, SII, and SIRI. The disease-specific survival (DSS) and progression-free survival (PFS) rates of the patients were compared by patient characteristics, including the NLR, PLR, SII, and SIRI.

### 2.3. Statistics

The characteristics of the systemic inflammatory markers are shown as both medians and means. Student’s t-test was used to compare continuous characteristics between the two groups. Pearson’s test was used to analyze the correlation between two continuous variables. DSS was considered the period between surgery and the date of the last follow-up, or cancer-specific death. PFS was considered the period between surgery and the date of recurrence, locoregional progression, metastasis, or death. The DSS and PFS rates were analyzed using Kaplan–Meier survival curves and compared statistically with the log-rank test. Age, sex, and all variables with significant prognostic values in the univariate analysis were subjected to multivariate analyses using the Cox proportional hazards model. A two-sided *p* < 0.05 was considered statistically significant. The potential nonlinear relationships between the continuous PLR and the survival outcomes were flexibly analyzed using a restricted cubic spline (RCS) with four knots [36]. The median value was used as a continuous predictor. Statistical analyses were conducted using SPSS 21.0 for Windows (IBM Corporation, Armonk, NY, USA) and R version 4.1.2.

## 3. Results

### 3.1. Patient Characteristics

The mean age of the patients was 55.1 ± 15.2 years, ranging from 18 to 90 years. The majority were male (64.3%) and had tongue cancer (74.3%). The distribution of the pathologic stage among the patients was as follows: stage I, 29.4%; stage II, 18.2%; stage III, 20.5%; and stage IV, 32.0% (Table 2). We calculated a mean platelet count of 241.45 ± 69.75 × 10^9^/L, a mean NLR of 2.58 ± 2.02, a mean PLR of 140.70 ± 61.26, a mean SII of 619.53 ± 494.38 × 10^9^/L, and a mean SIRI of 1.33 ± 1.64 × 10^9^/L (Table 3). An increase in the lymphocyte count was correlated with the platelet count (r = 0.33, *p* < 0.001) and the monocyte count (r = 0.22, *p* < 0.001) but not with the neutrophil count (r = 0.0022, *p* = 0.97) (Supplementary Figure S1). Additionally, an increase in PLR was correlated with NLR (r = 0.48, *p* < 0.001) (Supplementary Figure S2).

### 3.2. Correlation between Inflammatory Markers and Clinical Factors

We examined the correlation between systemic inflammatory markers and clinicopathologic parameters. The mean WBC count was higher in patients with a depth of invasion >1 cm and advanced T and AJCC stages (1 and 2 vs. 3 and 4) (all *p* < 0.05) (Supplementary Table S1). Platelet levels were higher in the younger patient group (*p* = 0.0284), but other markers showed no difference between the two age groups (Supplementary Table S1). There were no significant differences between the high and low NLR groups in the clinicopathologic parameters. High PLR was correlated with >1 cm depth of invasion and advanced T and AJCC stages (all *p* < 0.05). Likewise, advanced stage was correlated with high SII and SIRI (*p* = 0.0015 and 0.0131, respectively) (Table 4).

The survival analysis results are shown in Table 5. The median follow-up period was 36 months (range 0~185 months). Of the 269 patients, 65 patients died during the follow-up period, and 93 patients experienced disease progression. The 2-year and 5-year DSS rates of the OSCC patients were 78.8% and 75.6%, respectively.

**Figure 1:**
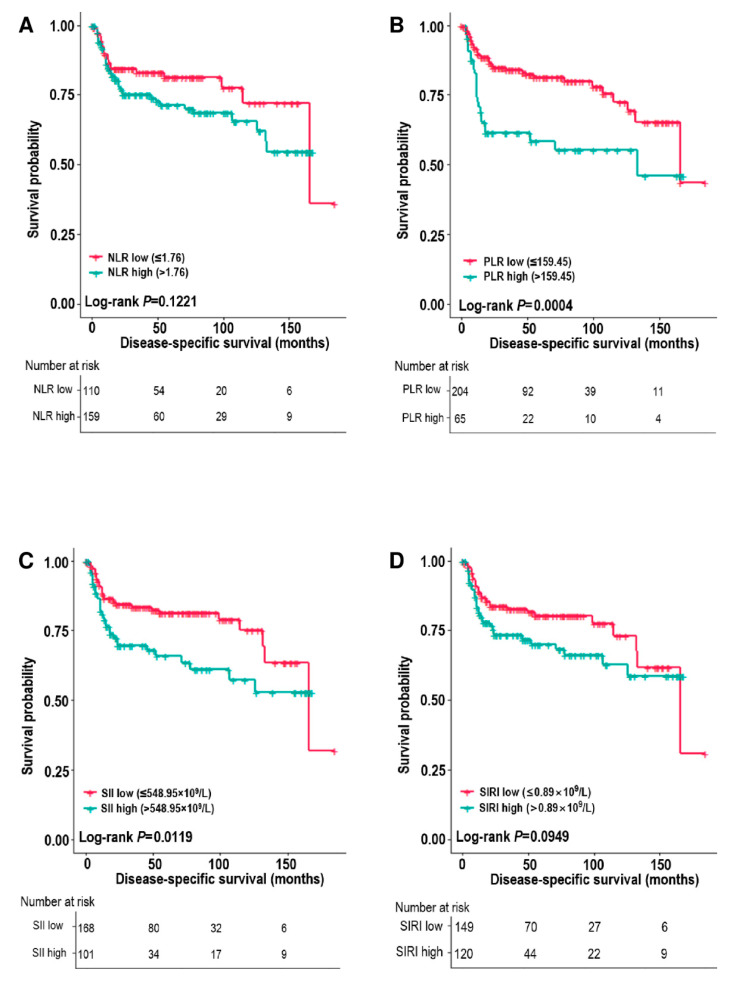
Disease-specific survival estimates (Kaplan–Meier) according to the neutrophil–lymphocyte ratio (NLR) (**A**), platelet–lymphocyte ratio (PLR) (**B**), systemic inflammation index (SII) (**C**), and systemic inflammation response index (SIRI) (**D**).

**Figure 2:**
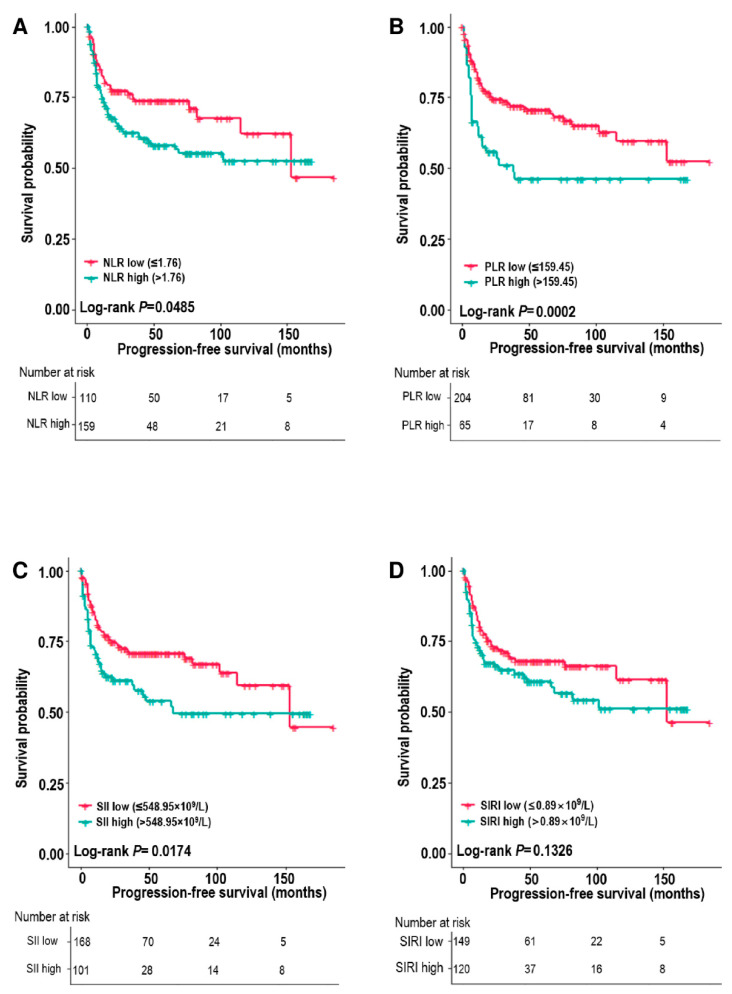
Progression-free survival estimates (Kaplan–Meier) according to the neutrophil–lymphocyte ratio (NLR) (**A**), platelet–lymphocyte ratio (PLR) (**B**), systemic inflammation index (SII) (**C**), and systemic inflammation response index (SIRI) (**D**).

Among the systemic inflammatory markers, the survival analysis revealed poorer DSS and PFS for patients with high PLR and SII (all *p* < 0.05) (Figure 1 and Figure 2). The 5-year DSS rates of the low vs. high PLR groups were 81.2% vs. 58.4% (*p* = 0.0004), and the 5-year PFS rates of the low vs. high PLR groups were 70.2% vs. 45.9% (*p* = 0.0002). NLR was associated with PFS (*p* = 0.0485), and SIRI showed no association with DSS or PFS (*p* = 0.012) (Figure 1 and Figure 2).

We entered factors that were significant in univariate analysis into the multivariate model, and the high PLR remained significant for DSS and PFS (DSS: hazard ratio (HR) = 2.36, 95% CI 1.28–4.36, *p* = 0.0059; PFS: HR = 1.80, 95% CI 1.06–3.06, *p* = 0.0300). Platelet count over 296.5 × 10^9^/L was not an independent prognostic factor for DSS and PFS (*p* = 0.141 and *p* = 0.531, respectively).

### 3.3. Analysis of the Relationship between PLR and Survival According to Clinical Factors

**Figure 3:**
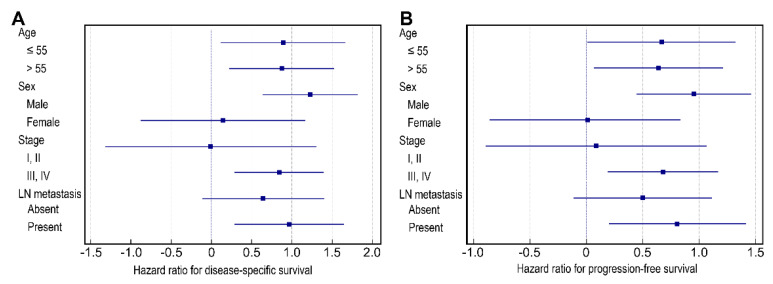
Forest plot of disease-specific survival hazard ratios (**A**) and progression-free survival hazard ratios (**B**) by subgroup. PLR, platelet–lymphocyte ratio; LN, lymph node.

We conducted subset analyses of the impact of PLR on survival according to selected clinicopathologic factors using forest plots. Notably, PLR was more strongly associated with DSS and PFS in patients who were male, had stage III/IV OSCC, or had lymph node metastasis (all *p* < 0.05) (Figure 3).

### 3.4. Nonlinear Association between PLR and Survival

**Figure 4:**
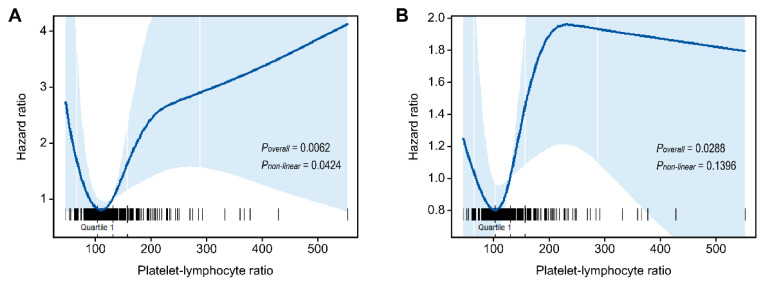
Hazard ratio for the risk of disease-specific death (**A**) and progression (**B**) in oral squamous cell carcinoma patients, evaluated by restricted cubic splines from unadjusted Cox proportional hazard models. The solid line represents the central risk estimate, and the shaded area represents the 95% confidence interval. The rug plot is of cases.

Furthermore, PLR was studied as a continuous variable in univariate analysis. The RCS analysis showed a curvilinear, J-shaped association between the PLR and survival outcomes rather than a straight line (Figure 4). This result suggested a possible nonlinear association between the PLR and the risk of DSS (*p*-value for nonlinearity = 0.0424). We did not find statistical evidence for nonlinearity with the progression-free survival outcome. We estimated the mortality risk to reach a nadir PLR in the range of 100–120, with inverse associations below that range and positive associations above that range, although the magnitude of associations varied.

## 4. Discussion

The inflammatory response is greatly influenced by tumor manifestation [37,38]. Many cancers cause extensive disruption of hematopoiesis [11]. In particular, cell mediation is closely associated with tumor development, growth, and metastasis [39]. There are methods to measure the inflammatory response, including C-reactive protein, erythrocyte sedimentation rate, and peripheral blood cell count [40,41]. NLR, PLR, and monocyte–lymphocyte ratio (MLR) are measured from peripheral blood cell counts, and illustrate how much neutrophils, platelets, and monocytes are increased compared to lymphocytes. These changes represent the cell-mediated systemic inflammatory response [42]. In studies conducted over the last decade, the presence of an elevated NLR and PLR has been associated with poorer outcomes in different types of malignancies [14,42,43]. Some researchers termed systemic inflammatory responses “the tip of the cancer iceberg” [44], but this approach is underutilized in clinical practice.

Recently, new prognostic scores or indexes were created through a combination of nutrition index, performance index, or three or more peripheral blood cell counts [23,45]. SII and SIRI are recently suggested novel prognostic biomarkers, derived from a combination of the absolute neutrophil count, lymphocyte count, and monocyte count. The prognostic value of each has been suggested in patients with lung cancer [24]. In our study, high SII and SIRI were associated with poor DSS in patients with OSCC in the univariate analysis. They were promising prognostic biomarkers in OSCC, but they failed to be identified as independent prognostic factors in the multivariate analysis.

In OSCC, several studies have investigated the prognostic value of NLR and PLR [33,46]. In a meta-analysis of 10 studies, a high NLR was associated with a poor prognosis in patients with OSCC [33]. PLR was also an independent prognostic factor in previous studies [46,47,48], and PLR was superior in the studies by Tazeen et al. [46] and Rosculet et al. [48]. In concordance with previous studies, our data showed that PLR was more strongly associated with overall survival and PFS than NLR, SII, and SIRI in patients with OSCC. NLR, SII, and SIRI were associated with either DSS or PFS in the univariate analysis, but PLR remained the only significant prognostic indicator after adjustments in the multivariate analysis. The subset analysis revealed that PLR had a greater prognostic impact in patients with advanced disease (stage III and IV) than in those with localized disease (stage I and II) (HR = −0.01, 95% CI −1.32 to 1.32, *p* = 0.9932 vs. HR = 0.84, 95% CI 0.29 to 1.40, *p* = 0.0028). This may be explained by the fact that cancer cells interact little with the microenvironment and local inflammatory cells in the low stages, and subsequently elicit little systematic inflammation [11,39,49]. Our data suggest that more active surveillance or treatment may be required for patients with high PLR, especially for those with stage III or IV OSCC. We need to actively seek alternative therapies such as immunotherapy or molecular targeted therapy in this patient group because their prognosis is predicted to be poorer after surgery.

Furthermore, we analyzed PLR as a continuous variable as well as a dichotomized variable. Interestingly, the prognostic correlation of PLR did not show monophasic linearity. The J-shaped relationship between DSS and PLR indicates an unfavorable prognosis for not only the high PLR group but also the extremely low PLR group. A J-shaped association is often observed in epidemiologic relationships, such as the association of body mass index and mortality [50]. A few researchers demonstrated a similar nonlinear association between NLR and prognosis in patients with gastric, breast, and oral cancers in the United States general population [51,52,53,54,55]. The nonlinear relationship between the survival outcome and pretreatment PLR has never been demonstrated previously. Our data showed that PLR has a nonlinear prognostic pattern similar to NLR. The increased risk of DSS at extremely low PLRs might indicate the strong cancer-related disturbance of normal hematopoiesis in these patients and its effect on survival, or the pervasive effect of unknown underlying noncancer-related health conditions. Nevertheless, an explicit rationale for such a pattern has yet to be established. It is necessary to further research the nonlinear prognostic pattern of PLR and to consider how to employ the best PLR thresholds in clinical practice.

The underlying mechanisms of PLR as a prognostic marker have not been fully elucidated, but there are several explanations. Bodies of evidence have shown that platelet activation is a key biological process for cancer occurrence, progression, and metastasis [56,57]. The phenomena related to platelet activation through interaction with cancer cells are thrombocytosis and thromboembolism [58]. Thrombocytosis is often observed in patients with advanced malignancy, and thromboembolism occurs frequently in cancer patients, with lethal consequences [57]. Aggregated platelets could enhance tumorigenesis by releasing pro-angiogenic mediators within the tumor microvasculature [59]. Furthermore, platelets could influence the metastatic potential of cancer cells through several biological pathways, i.e., secretion of cellular growth factors, helping stable tumor cell adhesion to endothelial cells, and impeding cell-mediated immunity against tumor cells [59,60,61,62,63,64]. A newly discovered link between platelets and cancer cells is tumor-educated platelets [65]. Tumor-educated platelets were most recently found through liquid biopsy research. Tumor-educated platelets are functional cells with a distinct tumor-driven phenotype that are thought to acquire tumor-derived factors and undergo signal-dependent changes in RNA processing within blood circulation [65]. Tumor-educated platelets have been shown to participate in multiple steps of metastasis, leading to lethal consequences [66,67]. There is a hypothesis that microvesicles containing RNA and proteins taken up by platelets promote tumor growth and immune evasion [68,69]. The relationship between tumor-educated platelets and PLR elevation is unclear. Future research on this matter would be interesting and would help elucidate the biological mechanisms underlying PLR as a biomarker.

The strength of our study is that various, not a single, systemic inflammatory markers were analyzed and that this was performed in a homogenous, rich sample group. Exploration of the nonlinear association between PLR and survival is another strength of this study.

However, our study had several limitations. First, its single-center, retrospective design may have caused potential bias. The systemic inflammatory marker cutoffs were derived from the AUC of these parameters against overall survival. Moreover, these cutoffs were not assessed in another data set for validation. Therefore, to use PLR in clinical practice, further prospective study or consensus on the optimal cutoff would be desirable. Secondly, our study could not set appropriate criteria for application, considering the major limitation of the systemic inflammatory markers, i.e., the influence of infection. Accumulation of scientific evidence and an understanding of the role of systemic markers through prospective multicenter trials would be needed to address this limitation.

In conclusion, this study evaluated the prognostic value of a panel of systemic inflammatory markers, including NLR, PLR, SII, and SIRI, in OSCC. Our data demonstrated that PLR was a valuable independent prognostic biomarker in patients with OSCC, especially in those with advanced disease. High PLR was associated with worse survival in these patients. We also demonstrated a nonlinear correlation between PLR and survival. A J-shaped association implied that an extremely low PLR was also a poor prognostic factor in patients with OSCC. Preoperative assessments of cellular biomarkers from peripheral blood could provide high-quality prognostic information, and they represent another promising approach for improving patient stratification.

## Figures and Tables

**Table 1:** Cutoff values of systemic inflammatory markers determined by receiver operating curves for overall survival.

	Cutoff Value	AUC	Sensitivity	Specificity	Accuracy
Platelet	296.5	0.5667	0.2923	0.8676	0.7286
NLR	1.7584	0.5407	0.6769	0.4363	0.4944
PLR	159.4521	0.5983	0.4	0.8088	0.71
SII, 10^9^/L	548.9451	0.5615	0.5077	0.6667	0.6283
SIRI, 10^9^/L	0.8938	0.5422	0.5385	0.5833	0.5725

AUC, area under the curve; NLR, neutrophil–lymphocyte ratio; PLR, platelet–lymphocyte ratio; SII, systemic inflammation index; SIRI, systemic inflammation response index.

**Table 2:** Clinicopathologic characteristics of patients with oral squamous cell carcinoma.

Characteristic	Number (%)
Total	269
Age (mean, years)	55.1 ± 15.2
Sex	
Male	173 (64.3%)
Female	96 (35.7%)
Location	
Mobile tongue	200 (74.3%)
Other (palate, lip, retromolar area, etc.)	69 (25.7%)
Tumor size (cm)	2.7 ± 1.7
Depth of invasion (cm)	1.0 ± 0.9
Differentiation	
Well	133 (49.4%)
Moderate	120 (44.6%)
Poor	16 (6.0%)
T stage	
T1	82 (30.5%)
T2	73 (27.1%)
T3	87 (32.3%)
T4	27 (10.0%)
N stage	
N0	146 (61.1%)
N1	22 (9.2%)
N2	23 (9.6%)
N3	46 (19.3%)
N4	2 (0.8%)
Stage	
I	79 (29.4%)
II	49 (18.2%)
III	55 (20.5%)
IV	86 (32.0%)
Adverse pathologic features	
Lymphatic invasion	73 (27.1%)
Vascular invasion	8 (3.0%)
Perineural invasion	77 (28.6%)
Adjuvant therapy	
Radiation therapy alone	56 (20.8%)
Chemotherapy and radiation therapy	60 (22.3%)
None	153 (56.9%)

**Table 3:** Summary statistics of inflammatory markers in patients with oral squamous cell carcinoma.

Parameter	Mean ± SD	Median (Range)	Cutoff Value	Population (*n* = 269) with Given Cutoff, Number (%)
Differential white blood cell count				
Neutrophil count, 10^9^/L	4.23 ± 2.14	3.64 (0.87–12.90)	NA	NA
Lymphocyte count, 10^9^/L	1.89 ± 0.67	1.82 (0.52–0.44)	NA	NA
Monocyte count, 10^9^/L	0.47 ± 0.19	0.42 (0.00–1.42)	NA	NA
Platelet count, 10^9^/L	241.45 ± 69.75	234.0. (37.20–652.00)	>296.5	46 (17.10%)
Calculated ratio and index				
NLR	2.58 ± 2.02	1.94 (0.37–16.00)	>1.76	159 (59.11%)
PLR	140.70 ± 61.26	130.99 (22.17–551.81)	>159.45	65 (24.16%)
SII, 10^9^/L	619.53 ± 494.38	452.42 (66.78–3515.33)	>548.95	101 (37.55%)
SIRI, 10^9^/L	1.33 ± 1.64	0.83 (0–16.04)	>0.89	120 (44.61%)

**Table 4:** Correlation between clinicopathologic parameters and systemic inflammation markers.

Parameter	No.	NLR High (>1.76)		PLR High (>159.45)		SII High (>548.95 × 10^9^/L)		SIRI High (>0.89 × 10^9^/L)	
		(*n* = 159)	*p*	(*n* = 65)	*p*	(*n* = 101)	*p*	(*n* = 120)	*p*
Age									
≤55	134	84 (52.8%)	0.2970	33 (50.8%)	0.9140	53 (52.5%)	0.5604	67 (55.8%)	0.0964
>55	135	75 (47.2%)		32 (49.2%)		48 (47.5%)		53 (44.2%)	
Sex									
Male	173	52 (32.7%)	0.2195	22 (33.9%)	0.7219	32 (31.7%)	0.2878	34 (28.3%)	0.0239
Female	96	107 (67.3%)		43 (66.2%)		69 (68.3%)		86 (71.7%)	
Location									
Mobile Tongue	200	116 (73.0%)	0.5292	45 (69.2%)	0.2779	73 (72.3%)	0.5462	84 (70.0%)	0.1427
Other	69	43 (27.0%)		20 (30.8%)		28 (27.7%)		36 (30.0%)	
Depth of invasion									
≤1 cm	165	93 (58.5%)	0.2489	30 (46.2%)	0.0039	55 (54.5%)	0.0723	66 (55.0%)	0.0554
>1 cm	104	66 (41.5%)		35 (53.9%)		46 (45.5%)		54 (45.0%)	
Lymphatic invasion									
Absent	196	112 (70.4%)	0.2828	45 (69.2%)	0.4496	65 (64.4%)	0.015	80 (66.7%)	0.0403
Present	73	47 (29.6%)		20 (30.8%)		36 (35.6%)		40 (33.3%)	
Vascular invasion									
Absent	261	105 (95.5%)	0.2783	198 (97.1%)	0.9553	99 (98.0%)	0.7141	119 (99.2%)	0.0789
Present	8	5 (4.6%)		6 (2.9%)		2 (2.0%)		1 (0.8%)	
Perineural invasion									
Absent	192	85 (77.3%)	0.0751	150 (73.5%)	0.1662	66 (65.3%)	0.0904	74 (63.3%)	0.0090
Present	77	25 (22.7%)		54 (26.5%)		35 (34.7%)		44 (36.7%)	
T stage									
T1 and T2	155	88 (55.4%)	0.3640	27 (41.5%)	0.0026	52 (51.5%)	0.1143	62 (51.7%)	0.0761
T3 and T4	114	71 (44.7%)		38 (58.5%)		49 (48.5%)		58 (48.3%)	
Lymph node metastasis									
Absent	176	75 (68.2%)	0.4295	40 (61.5%)	0.4490	60 (59.4%)	0.1074	72 (60.0%)	0.0930
Present	93	35 (31.8%)		25 (38.5%)		41 (40.6%)		47 (40.0%)	
Stage									
I, II	128	69 (43.4%)	0.0983	22 (33.9%)	0.0109	37 (36.6%)	0.0053	47 (39.2%)	0.0131
III, IV	141	90 (56.0%)		43 (66.2%)		64 (63.4%)		73 (60.8%)	
Distant metastasis									
Absent	254	152 (95.6%)	0.3132	60 (92.3%)	0.3932	95 (94.1%)	0.8400	115 (95.8%)	0.3659
Present	15	7 (4.4%)		5 (7.7%)		6 (5.9%)		5 (4.2%)	

NLR, neutrophil–lymphocyte ratio; PLR, platelet–lymphocyte ratio; SII, systemic inflammation index; SIRI, systemic inflammation response index.

**Table 5:** Survival analysis of patients with oral squamous cell carcinoma according to clinicopathologic parameters and systemic inflammatory markers.

Variables	Disease-Specific Survival			Progression-Free Survival		
	Univariate Analysis	Multivariate Analysis		Univariate Analysis	Multivariate Analysis	
	*p*	*p*	Hazard Ratio (95% CI)	*p*	*p*	Hazard Ratio (95% CI)
Age (>55 years)	0.1293	0.2252	1.38 (0.82–2.34)	0.1329	0.3500	1.23 (0.79–1.92)
Gender (Male)	0.2874	0.9221	0.97 (0.54–1.73)	0.3462	0.7593	0.93 (0.58–1.48)
T stage (reference 1)	<0.0001	0.8286		<0.0001	0.6317	
2		0.6694	0.62 (0.07–5.63)		0.9388	0.92 (0.11–7.81)
3		0.4558	0.39 (0.03–4.70)		0.8899	1.18 (0.12–11.61)
4		0.6402	0.54 (0.04–7.15)		0.5508	2.04 (0.20–21.25)
N stage (reference 0)	<0.0001	0.6277		<0.0001	0.9787	
1		0.1091	0.32 (0.08–1.29)		0.6247	0.77 (0.27–2.17)
2		0.6264	0.69 (0.15–3.13)		0.6875	0.77 (0.21–2.78)
3		0.6979	0.76 (0.18–3.12)		0.8974	0.92 (0.27–3.10)
4		0.9726	0.00 (0–1000)		0.9609	0.00 (0–1000)
DOI (>1 cm)	<0.0001	0.4120	1.75 (0.46–6.60)	<0.0001	0.7644	0.86 (0.32–2.33)
Stage (reference I)	<0.0001	0.3739		<0.0001	0.7194	
II		0.4611	2.53 (0.22–29.72)		0.7179	1.52 (0.16–14.96)
III		0.2311	4.22 (0.40–44.57)		0.5945	1.83 (0.20–16.92)
IV		0.1079	8.59 (0.62–118.32)		0.3427	3.19 (0.29–34.96)
Lymphatic invasion	0.00086	0.1813	1.56 (0.81–2.98)	0.0010	0.0757	1.64 (0.95–2.84)
Vascular invasion	0.7050	-	-	0.6535	-	-
Perineural invasion	<0.0001	0.1651	1.51 (0.84–2.71)	0.0030	0.9179	1.03 (0.62–1.70)
Differentiation (reference well)	0.0022	0.0663		0.0025	0.1059	
Moderate		0.4403	0.80 (0.46–1.41)		0.8445	1.05 (0.66–1.65)
Poor		0.0622	2.33 (0.96–5.67)		0.0380	2.28 (1.05–4.97)
Platelet high	0.0013	0.1474	1.60 (0.84–3.00)	0.0093	0.5314	1.20 (0.68–2.13)
NLR high	0.1221	-	-	0.0485	0.4553	1.25 (0.69–2.27)
PLR high	0.0004	0.0064	2.33 (1.27–4.28)	0.0020	0.0300	1.80 (1.06–3.06)
SII high	0.0119	0.6822	0.88 (0.46–1.65)	0.0174	0.5836	0.83 (0.44–1.59)
SIRI high	0.0949	-	-	0.1326	-	-

CI, confidence interval; NLR, neutrophil–lymphocyte ratio; PLR, platelet–lymphocyte ratio; SII, systemic inflammation index; SIRI, systemic inflammation response index. In univariate analysis, T stage, N stage, depth of invasion, AJCC stage, lymphatic invasion, perineural invasion, tumor differentiation, and platelet count were associated with DSS and PFS (all *p* < 0.05).

## Data Availability

The data presented in this study are available on request from the corresponding author.

## Supplementary Materials

The following supporting information can be downloaded at: https://www.mdpi.com/article/10.3390/biomedicines10061268/s1, Figure S1: Pearson’s correlation between lymphocyte count and platelet count, neutrophil count, and monocyte count; Figure S2: Pearson’s correlation between platelet-lymphocyte ratio and neutrophil-lymphocyte ratio; Table S1: Correlation between clinicopathologic parameters and white blood cell and platelet count.
