# Supplementary material for: Prognostic Role of Systemic Inflammatory Markers in Patients Undergoing Surgical Resection for Oral Squamous Cell Carcinoma
Source: Biomedicines. 2022 May 29;10(6):1268. doi: 10.3390/biomedicines10061268 (PMC9220324; doi:10.3390/biomedicines10061268)
Supplement: Supplementary file 1 [file biomedicines-10-01268-s001.zip › Supplementary Figure S1.pdf]

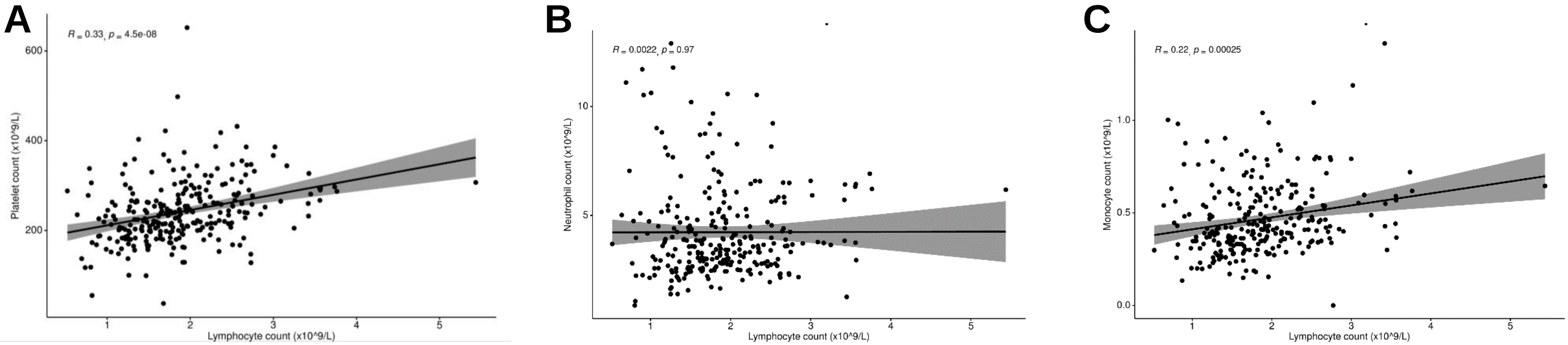

Supplementary Figure S1. Pearson's correlation between lymphocyte count and (A) platelet count, (B) neutrophil count, and (C) monocyte count
