# Supplementary material for: Prognostic Role of Systemic Inflammatory Markers in Patients Undergoing Surgical Resection for Oral Squamous Cell Carcinoma
Source: Biomedicines. 2022 May 29;10(6):1268. doi: 10.3390/biomedicines10061268 (PMC9220324; doi:10.3390/biomedicines10061268)
Supplement: Supplementary file 1 [file biomedicines-10-01268-s001.zip › Supplementary Figure S2.pdf]

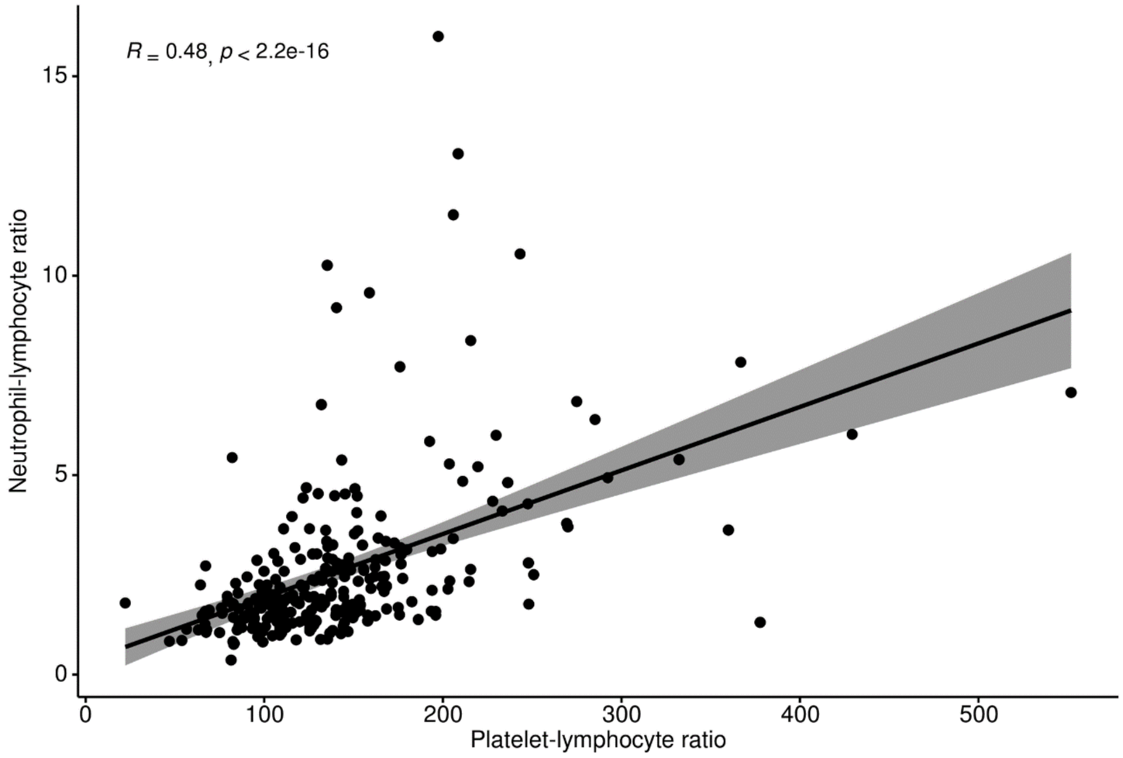

Supplementary Figure S2. Pearson’s correlation between platelet-lymphocyte ratio and neutrophil-lymphocyte ratio.
