# Supplementary material for: Prognostic Role of Systemic Inflammatory Markers in Patients Undergoing Surgical Resection for Oral Squamous Cell Carcinoma
Source: Biomedicines. 2022 May 29;10(6):1268. doi: 10.3390/biomedicines10061268 (PMC9220324; doi:10.3390/biomedicines10061268)
Supplement: Supplementary file 1 [file biomedicines-10-01268-s001.zip › Supplementary Table S1.pdf]

Supplementary Table S1. Correlation between clinicopathologic parameters and white blood cell and platelet count

Parameter	No.	WBC (mean ± SD)	*P* *	Platelet count (mean ± SD)	*P* *
**Age**					
≤55	134	6.76 ± 2.33	0.9863	250.79 ± 68.11	0.0284
>55	135	6.77 ± 2.40		232.18 ± 70.37	
**Sex**					
Male	173	6.95 ± 2.43	0.0942	238.90 ± 71.78	0.4217
Female	96	6.44 ± 2.22		246.04 ± 66.06	
**Location**					
Tongue	200	6.67 ± 2.31	0.2442	245.09 ± 73.34	0.1455
Other	69	7.05 ± 2.49		230.90 ± 57.29	
**Depth of invasion**					
≤1 cm	165	6.53 ± 2.20	0.0438	235.93 ± 58.58	0.1316
>1 cm	104	7.14 ± 2.56		250.20 ± 84.08	
**Lymphatic invasion**					
Absent	196	6.60 ± 2.27	0.0565	237.38 ± 63.18	0.1711
Present	73	7.22 ± 2.56		252.36 ± 84.45	
**Vascular invasion**					
Absent	261	6.79 ± 2.38	0.3168	241.46 ± 70.42	0.9813
Present	8	5.94 ± 1.72		240.88 ± 45.73	
**Perineural invasion**					
Absent	192	6.66 ± 2.34	0.2530	241.91 ± 69.79	0.8634
Present	77	7.03 ± 2.42		240.29 ± 70.10	
**T stage**					
T1&T2	155	6.46 ± 2.15	0.0166	234.65 ± 56.79	0.0785
T3&T4	114	7.18 ± 2.57		250.69 ± 83.63	
**Lymph node metastasis**					
Absent	176	6.67 ± 2.32	0.3466	240.49 ± 66.82	0.7586
Present	93	6.95 ± 2.45		243.25 ± 75.32	
**Stage**					
I, II	128	6.36 ± 2.04	0.0062	234.49 ± 57.20	0.1139
III, IV	141	7.14 ± 2.57		247.76 ± 79.13	
**Distant metastasis**					
Absent	254	6.78 ± 2.34	0.7242	241.25 ± 69.79	0.8514
Present	15	6.56 ± 2.83		244.73 ± 71.40	

*Student's t test was used for the comparison of means. SD, standard deviation; WBC, white blood cell
